# Association between highly active antiretroviral therapy (HAART) and hypertension in persons living with HIV/AIDS at the Bamenda regional hospital, Cameroon

**DOI:** 10.11604/pamj.2019.33.87.15574

**Published:** 2019-06-06

**Authors:** Pepanze Jill Pangmekeh, Mbunka Muhamed Awolu, Simo Gustave, Tayong Gladys, Samuel Nambile Cumber

**Affiliations:** 1 University of Dschang, Department of Biomedical Sciences, Dschang, Cameroon; 2 Regional Coordinator For the Fight Against HIV/AIDS North West Region, Bamenda, Cameroon; 3 Faculty of Health Sciences, University of the Free State, Bloemfontein, South Africa; 4 Section for Epidemiology and Social Medicine, Department of Public Health, Institute of Medicine, The Sahlgrenska Academy at University of Gothenburg, Box 414, SE-405 Gothenburg, Sweden; 5 School of Health Systems and Public Health, Faculty of Health Sciences, University of Pretoria Private Bag X323, Gezina, Pretoria, 0001, Pretoria, South Africa

**Keywords:** Hypertension, HAART, HAART-naïve, association, PLWHIV

## Abstract

**Introduction:**

The introduction of highly active antiretroviral therapy (HAART) in the treatment of HIV infection has provided different good results: like long-term viral suppression, the decrease of opportunistic infections, and repair of the immune system.

**Methods:**

We carried out a hospital-based cross-sectional analytic study involving 315 participants 228 were on HAART (group 1) and 87 were HAART-naïve (group 2) at the HIV treatment centre of the Bamenda regional hospital with our study population being all people living with HIV (PLWHIV) in the North West region of Cameroon. The sampling was performed from the 15^th^ of March to the 30^th^ of June 2017. The questionnaire was administered face to face with participants and their vital signs taken. Blood pressure was measured using an automated electronic blood pressure monitor and hypertension (HTN) was considered as systolic blood pressure (BP) ≥ 140 mmHg and/or diastolic BP ≥ 90mmHg.

**Results:**

The prevalence of hypertension in the HAART group was 36.44% (n=82, CI: 30.15%-43.10%) compared to that of the HAART-naïve group which was 13.33% (n=12, CI: 7.08%-22.13%, P=0.01). HAART was associated with HTN after controlling for gender, family history of hypertension, body mass index (BMI), smoking and alcohol consumption. The odds ratio of the HAART-treated versus the HAART-naïve was 3.86 (95% CI: 1.98-7.50). We also found an association between TDF/3TC/EFV (OR=2.83), AZT/3TC/NVP (OR=2.82), AZT/3TC+EFV (OR=3.48) and TDF/3TC+NVP (OR=2.36) and HTN whereas those on AZT+3TC+ATV/r (OR=0.84) and TDF+3TC+ATV/r (OR=0.45) were not associated to hypertension.

**Conclusion:**

Our result suggests that blood pressure should be periodically measured and treated when necessary in PLWHIV on HAART.

## Introduction

HIV is a major public health problem in the world today. In 2015, 2.1 million new HIV infections were recorded worldwide, adding up to a total of 36.7 million people living with HIV in the world [1]. About 1.1 million people died of AIDS-related illnesses worldwide [1]. Although the burden of the epidemic continues to vary considerably between countries and regions, sub Saharan Africa remains the most severely affected with 4.4% of persons living with HIV [1]. This percentage accounts for nearly 70% of the people living with HIV worldwide. Countries in North Africa and the horn of Africa have a significantly lower prevalence rates as their populations typically engage in fewer high risk cultural patterns that have been implicated in the spread of the virus in sub Saharan Africa (SSA) [1]. The number of people living with HIV in Cameroon is estimated to about 620,000 (UNAIDS). Adults aged 15 and above living with HIV are 580,000, adults aged from 15-49 have a prevalence rate of 4.5%. Women aged 15 and above living with HIV is 340,000 and the number of death due to AIDS in Cameroon is 33,000 [1]. Since the introduction of Highly Active Anti-retroviral Therapy (HAART), in the mid-1990s, the number of people living with HIV and under antiretroviral therapy has increased by about a third in the last 2 years; reaching 17.0 million people compared to the 15 million that was targeted in 2015 by the United Nations General Assembly in 2011. Since the first global treatment target was set in 2003, annual AIDS-related deaths have decreased by 43% [1]. In the world's most affected region, eastern and southern Africa, the number of people on treatment has more than doubled since 2010, reaching nearly 10.3 million people. AIDS related deaths in the region have decreased by 36% since 2010 [1]. HIV-related morbidity and mortality rates have dropped remarkably since the introduction of HAART. Whereas concerns of a link between HAART and coronary heart disease (CHD) have increased. An elevated risk of myocardial infarction among people taking HAART has been found in some studies. Investigators have assessed the relationship between the uses of antiretroviral therapy (ART) and components of the metabolic syndrome including dyslipidemia [2], insulin resistance [1, 2] and abnormal fat distribution [3]. Despite the scarcity of data on cardiovascular diseases (CVD) in people living with HIV in Africa, a study estimates the prevalence of self-reported CVD risk factors in HIV patients in Africa at 12% [4].

In Uganda, about 18% of HIV-infected adults were found with sub-clinical atherosclerosis which can be predictive of CVD [5]. Recent data suggest that cardiovascular diseases such as heart failure, coronary artery diseases hypertension and stroke are common and appear to be very frequent in HIV infected population in low and middle income countries [5, 6]. HAART and especially protein inhibitors (PIs) seem to be associated with metabolic dysfunction and could increase the risk of cardiovascular events [6]. Recent reports raise increasing suspicion that HAART may also induce hypertension [7-11]. Conflicting results have been reported for the association between HAART and blood pressure and for the association between HAART and the prevalence of hypertension [12, 13]. The relationship between HAART and hypertension has not been well studied [13]. Hypertension appears to be linked to insulin resistance; in particular, hypertension seems to be a part of the metabolic syndrome [14]. Interestingly, increase blood pressure has been found to be associated with lipodystrophy [13]; providing thus additional evidence that HAART and hypertension may be linked via pathways involving lipodystrophy or other metabolic disorders [15]. The association between HAART and hypertension has been documented elsewhere [7-13]. Due to the conflicting results between HAART and hypertension and the lack of data on this subject in the North West region of Cameroon, there is a need to improve our knowledge on the impact of HAART on the possible development of side effects such as hypertension in people living with HIV (PLWHIV) and under ART. This study was carried out with the aim to identify side effects resulting from ART in order to improve the control of HIV/AIDs by putting at the disposal of policy makers and stakeholders' information that could help them to ameliorate the treatment of PLWHIV.

**Research question:** how can the identification of side effects resulting from HAART help in the management of PLWHIV who are under treatment (HAART)

**Objective:** the main objective of this study was to evaluate blood pressure (hypertension) in PLWHIV and to determine the association between highly active antiretroviral therapy and hypertension in PLWHIV at the Bamenda Regional Hospital.

**The specific objectives were:** determine the prevalence of hypertension in PLWHIV under HAART and those not treated who attend the Bamenda regional hospital; compare the prevalence of hypertension in these two groups (PLWHIV under HAART and HAART-naïve clients) of PLWHIV; to determine the association between HAART and hypertension in PLWHIV.

## Methods

**Study design:** this was a hospital based cross-sectional analytic study at the HIV treatment centre of the Bamenda Regional Hospital. Our study population was all PLWHIV in the North West region. The study was conducted from the 15^th^ of March to the 30^th^ of June 2017.

**Study area:** the study was conducted in the Bamenda Regional Hospital's HIV (BRH's) treatment centre, a centre that provides longitudinal care to about 6400 PLWHIV a year for routine HIV care including follow up by doctors and other health personnel. The centre receives people between 7:30 a.m. to 4 p.m. from Monday to Friday. The centre has a waiting room equipped with benches where clients first wait upon arrival at the centre, a counselling room, a room to open files for new cases which is just beside the counselling room, a laboratory for HIV test, CD4, 4 rooms for data collection and two consultation rooms where clients are consulted and counselled. This treatment centre is located just beside the North West Regional Fund for Health promotion.

**Study population:** the study population was made up of PLWHIV of the North West region that visited the Bamenda Regional Hospital's treatment centre from March 2017 to June 2017.

**Target population:** for this study, the studied population was divided into two groups; the control group constituted of PLWHIV who had never received HAART (HAART-naïve), it is a group of all new cases of PLWHIV tested positive at the Bamenda Regional hospital from March 2017 to June 2017; the intervention groups who are PLWHIV on HAART people of this group were selected using randomized sampling techniques matched by age and gender to the HAART-naïve group.

### Selection criteria

**Inclusion criteria:** for the control group, all newly diagnosed cases in the Bamenda Regional Hospital aged 20 years and above who are not yet on treatment and consented to take part in the study were included in the study. For the intervention group, PLWHIV age 20 years and above who had been on continuous HAART for at least 24 months (2years) were included.

**Exclusion criteria:** all clients with pre-existing hypertension before HAART initiation whether on anti-hypertensive medication or not were excluded. Clients on oral contraceptives, corticosteroid and other medications whether traditional or modern medication that could affect blood pressure were also excluded. Clients with a family history of hypertension, with a confirmed non-adherence to HAART for 6 months, with confirmed psychological stress or any abnormal physical stress were also excluded. Those who smoke or drink more than 2 bottles of beer a week and who did not give their consent where also excluded.

**Sampling method and sample size:** the HIV treatment centre of the Bamenda Regional Hospital was chosen firstly for convenient reasons and also because this treatment centre provides care to the largest number of PLWHIV in the North West Region. People of the first group or PLWHIV who had never received HAART before (HAART-naïve) were recruited as they come to open their files to start treatment. During the opening of the file, they were sensitized on the on-going research activities which are performed in the centre by giving them their objectives and importance. The interested ones were sent to the principal investigator for more detail and clear explanations of the research objectives. Those who gave their informed consent were interviewed and physically examined. PLWHIV and who were on HAART or the second group were met in the waiting hall of the treatment centre where they assemble every morning for information, education, and communication. To this group, the objectives and importance of the research were explained. Those who were interested to participate in the study were directed to one of the treatment centre rooms where details and deep explanations were provided to them. As for the first group, those who gave their informed consent were interviewed and physically examined. After a face-to-face interview and a physical examination of each client who has given its informed consent and who are enrolled in the study, data were collected using a pre-tested structured questionnaire. Information on socio-demographics, smoking habits, alcohol consumption, family history of HTN, duration of HAART was obtained both from the interview and patients medical records. The physical examination entailed measurement of height, weight, body mass index (BMI), waist and hip circumferences and waist-to-hip ratio (WHR). The blood pressure of each participant was measured and hypertension (the outcome variables) was diagnosed from the BP value. Sample size: the minimum sample size (N) was determined using the formula.

$$N=\frac{{\text{Z}}^{2}*\text{P}\left(1-\text{P}\right)}{{\text{I}}^{2}}$$

Where N=minimum sample size, Z=reduce standard deviation for a margin of error α of 0.05, P= prevalence of hypertension in PLWHIV, I= statistic precision fixed at 0.05 Using pre-estimates of the prevalence of hypertension in PLWHIV of 28.5% (Akem *et al.* 2016) we thus needed 313 participants. To account for potential non-response, 344 participants were selected. Thus,

$$N=\frac{{\left(1.96\right)}^{2}*\left(0.285\right)\left(1-0.285\right)}{0.05^{2}}$$

Non-respondent was considered to be 10% of the sample size. This gives us a total of N=344 persons.

### Data management and statistical analysis

**Data quality assessment:** each filled questionnaire was checked again to ensure that all the questions are answered and correctly. All the questionnaires were checked daily for completeness and consistency. Those that were not correctly filled were rejected. The completed questionnaires were rechecked to maintain the quality of data.

**Statistical analyses:** at the end of each day, questionnaires were collected and coded before being transmitted for data entry. Double entries were made and analyzed using EPI Info 7 and Excel 2010. Descriptive and inferential statistical analysis was used. Uni-variate and multi-variate logistic regression analysis were used to determine if there is an association between HAART and hypertension. Unadjusted and adjusted odd ratio (AOR) and their corresponding 95% confidence interval were used to examine the strength of association. P values of less or equal to 0.05 was considered significant.

**Ethical consideration:** the protocol for this study was reviewed and approved by the University of Dschang; more so, authorization to carry out the research was obtained in the research site. Patient's confidentiality was respected and consented participants enrolled in to the study.

## Results

**Socio-demographic and clinical characteristics of participants:** a total of 315 participants were enrolled in the study, 228 were on HAART (group 1) and 87 were HAART-naïve (group 2). Participants age range from 22 to 75 years while their mean age was 44.129 (± 10.40) years. The corresponding age range for group 1 and group 2 were 22 to 75 years and 22 to 65 years while their mean age was 45.07(±10.70) and 41.62(±9.16) respectively (P=0.0085). Sixty-eight percent of the respondents (214), were aged between 36 to 55 years. The proportion of females were 74.67% (n=168) and 65.56% (n=59) in group 1 and group 2 respectively. The duration of HAART in group 1 ranged from 2 to 17 years. The mean BMI for all the participants was 25.99(±6.31), 33.97% (n=107) were overweight, 8.25% (n=26) had grade 1 obesity and 6.98% (22) had grade 2 obesities. In group 1, 9.78% (6.23% to 14.43%) were obese and 8.44% (5.16% to 12.87%) had grade 2 obesities while for group 2, 4.44% (1.22% to10.90%) were obese and 3.33% (0.69% to 9.43%) had grade 2 obesities. Generally, 74.60% (n=235) of the participants were married and 22.22% (n=70) were single, 76.51% (n=244) were self-employed while 17.46% (n=55) were employed. Body mass index was associated to hypertension. There was no significant difference between HAART and HAART-naïve group with respect to age, gender occupation and level of education. There was a significant difference (P=0.04) between HAART and HAART-naïve group with respect to BMI (Table 1).

**Table 1:** Socio-demographic and clinical characteristics of participants

Variables		General population	HAART group	HAART-naïve group
Sex n(%)	Male	88(18.47%)	57(25.45%)	31(34.44%)
	Female	226(71.97%)	167(74.55%)	59(65.56%)
Marital status n(%)	Married	235(74.60%)	170(75.56%)	65(72.22%)
	Single	70(22.22%)	48(21.33%)	22(24.44%)
Age (mean ± SD in years)		44 ±10.40	45.07±10.70	41.62±9.16
Profession, n (%)	Self-employed	241(76.51%)	172(76.44%)	69(76.67)
	Employed	55(17.46)	39(17.33%)	16(17.78%)
Prevalence of hypertension (n)		29.84%(94)	36.44%(82)	12 (13.33%)
Duration of HAART in years, n (%) <5		43(13.65%)	43(13.65%)	0
5<10		100(31.75%)	100(31.75%)	0
10<15		74(23.49%)	74(23.49%)	0
>15		8(2.54%)	8(2.54%)	0
BMI(Kg/m2)(Mean ± SD)		25.99±6.31	26.44±5.66	24.83±7.68
WHR(Mean ±SD)		1.98±4.19	2.08±5.65	1.73±0.45

**Figure 1:**
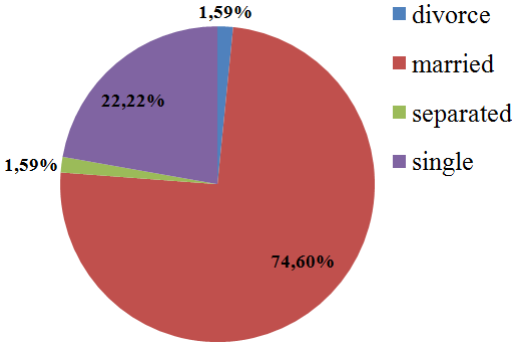
Marital status of all the study participants

**Marital status of participants:** 74.60% (n=235) of all the participants were married, 22.22% (n=70) were single, 1.59% (n=5) were separated and 1.59% (n=5) were divorce (Figure 1).

**Figure 2:**
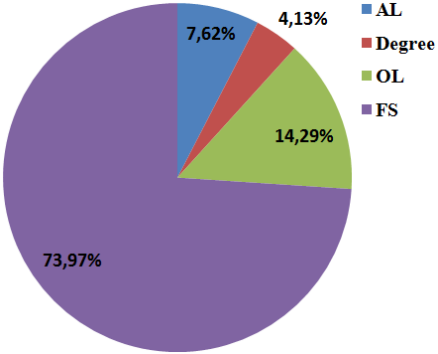
Level of education of study participants

**Level of education in all the study participants:** the level of education in all the participants with the greatest percentage having first school living certificate, 73.97% (n=233), followed by 14.29% (n=45) for those with ordinary level certificate, 7.62% (n=24) for those with advance level certificate and 4.13% (n=13) for those with bachelor's degree (Figure 2).

**Figure 3:**
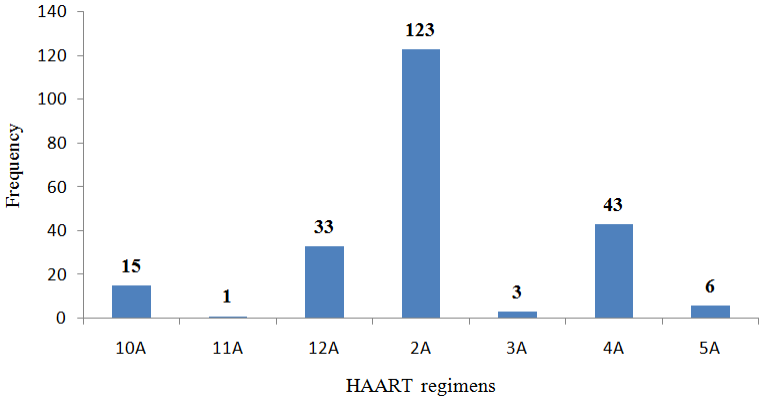
HAART regimens and the number of participants who ever received either of the regimens

**HAART regimens and the number of participants who ever received either of the regimens:** the various HAART regimens and the number of participants in each regimen. All the subjects on treatment were on a backbone of Lamivudine (3TC), 54.91% (n=123) on Tenofovir/Lamivudine/Efavirenz (2A), 19.20%(n=43 on, Zidovudine/Lamivudine/Nevirapine (4A), 14.73% (n=33) were Tenofovir/Lamivudine/Atanzanavir/Ritonavir(12A), 6.70% (n=15) on Zidovudine/Lamivudine/Atazanavir/Ritonavir(10A) and 2.68%(n=6) on Tenofovir/Lamivudine/Nevirapine (Figure 3).

**Figure 4:**
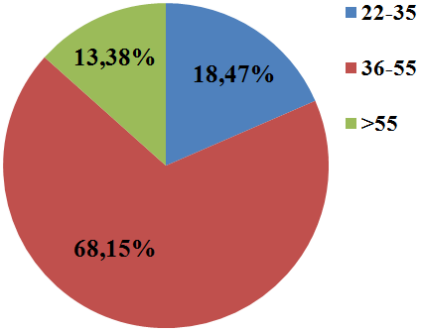
Age distribution of study participant

**Age distribution in the study participants:** the majority of the participant age ranges between the ages of 36 to 55 years (68.15%). Figure 4 shows the age distribution of all the participants.

**Prevalence of hypertension in the study participants:** the prevalence of hypertension in all the study participants was 29.84% (n=94, CI: 25.06%-35.11%). The prevalence of hypertension in the HAART group was 36.44% (n=82, CI: 30.15%-43.10%) and that of the HAART-naïve group was 13.33% (n=12, CI: 7.08%-22.13%). A significant difference (P=0.01) between the HAART and the HAART-naïve group was found (P=0.01) (Table 2). The highest prevalence of hypertension in the different HAART regimens was detected in people on Tenoforvir/Lamivudine/Efavirenz, (61.79%) and the lowest was identified in people on Zidovudine/Lamivudine/Efavirenz. TDF/3TC/EFV, AZT/3TC+EFV, AZT/3TC/NVP and TDF/3TC+NVP were significantly associated to hypertension in all the participants (Table 2).

**Table 2:** Prevalence of hypertension in the different HAART regimens

HAART regimens	Number	Percentage (%)	Odd Ratio
TDF/3TC/EFV	123	61.79	2.83
AZT/3TC+EFV	3	1.34	3.48
AZT/3TC/NVP	43	19.20	2.829
TDF/3TC+NVP	6	2.68	2.359
AZT+3TC+ATV/r	15	6.70	0.844
TDF+3TC+ATV/r	33	14.73	0.451

**Association between HAART and hypertension in PLWHIV:** in our analysis using logistic regression, we observed that being on HAART was significantly and positively associated with having HTN. The odd ratio (OR) of having hypertension comparing the HAART to the HAART-naïve group was 3.86 (95% CI: 1.98-7.50, P=0.01). Prevalence of hypertension in the HAART and HAART-naïve group; HAART (36.44%); HAART-naïve group (13.33%).

## Discussion

This study found a significant higher prevalence of hypertension among PLWHIV on HAART than their HAART-naïve counterparts. HAART was significantly and positively associated to hypertension even after adjusting for confounders. Trends in published research findings on this subject have shown a mixture of association and lack of association between HAART use and hypertension. This study is similar with that of several studies which show a higher prevalence of HTN with HAART [14]. This result supports the fact that HAART can possibly induce hypertension in people under HAART. Our results are not in line with other studies which revealed no significant difference in the prevalence of HTN between patients on HAART and HAART-naïve people [16] or even a lower prevalence of HTN with NNRTI's use [10]. These differences observed between results of these studies could be due to several factors such as differences in geographical location, study settings, clinical characteristics of the study participants and even study designs. The prevalence of hypertension in our participants on HAART (36.44%) was higher than that found in previous studies [16, 17]. This high prevalence can be explained by the fact that participants of our study were on HAART for a long time (7.92 years) compared to the participants of the above studies. This hypothesis is supported by the findings of the Multi-centre AIDs cohort study which suggested a link between the duration of HAART and high blood pressure [18]. This Multi-centre study showed, that prolong HAART (defined as 2-5years in duration) was independently associated with development of HTN, whereas HAART of less than 2 years in duration was not [18]. Contrary to our study which was predominantly composed of females, all the participants of the multi-centre AIDs cohort study were males. It is worth nothing that no participants received a definitive diagnosis of hypertension based on the measurements of blood pressure from our study. Those with high blood pressure were referred to the attending physician of the health care facility for further investigation. The prevalence of hypertension in our study population was similar but not in total to the findings of [19] carried out in South West region of Cameroon. This can be explained by the fact that the majority of the participants enrolled in the two studies were females or from common geographical locations. Controversial and conflicting association between antiretroviral therapy and systematic hypertension has been documented elsewhere [14, 16, 18, 19]. This first cross-sectional study investigating the influence of HAART on hypertension in HIV infected persons of the North West region (NWR) Cameroon revealed a systemic hypertension in 29.84% of our study participants having a mean age of about 44 years. In a multi-centre AIDS cohort study carryout by Serberg *et al*. [18] found similar risk of systolic hypertension in men on HAART and those who are HIV-negative during the first 2 years following the initiation of HAART. However, in the same multi-centre cohort study, these authors reported that a longer duration on HAART can substantially increase the risk of systolic hypertension in men. No change was seen in the diastolic blood pressure (DBP) [18]. In our study, the limited time of exposure to HAART was 2 years which might have influenced the blood pressure as predicted by the study carried out by Serberg *et al*. in the 2005 group [18] of hypertensive and normotensive patients had similar risk factors for age, gender and BMI.

A few cross-sectional studies have evaluated the effect of HAART on blood pressure [16]; compared the prevalence of hypertension in HIV-infected persons, naïve to HAART or receiving HAART and HIV-negative controls in a cross-sectional analysis. They reported the prevalence of hypertension to be 13% in the HAART-naïve persons, 21% in individuals receiving HAART and 24% in HIV-negative individuals. They concluded that the prevalence of hypertension in patients receiving HAART was not statistically different from that of the HIV-negative controls or treatment naïve patients [14, 16] looked at the prevalence of hypertension in relation to the metabolic syndrome in 287 HIV-positive persons receiving HAART and 287 age and gender matched controls. Compared to the control group, (2003) the authors found an increase prevalence of HTN (34.2% against 11.9%.p.value =0.0001) in participants on HAART. In another study where Crane *et al*. (2006) followed a cohort of 444 HAART-naïve, HIV-positive persons initiating different treatment regimens (PI-based regimens and NNRTI regimens) with a mean age 35 years and 84% of males, no significant difference was found between classes of drugs and blood pressure elevation. In this study, the cohort experienced a significant increase in mean SBP after initiating HAART (124.6 against 121.6 P<= 0.001). This author concluded that, patients receiving Lopinavir/ritonavir-based regimen, had a twofold increased risk of developing elevated blood pressure which was partly mediated by an increase in BMI. Our study showed a positive association (OR=3.86, 95% CI: 1.98%- 7.50%) between HAART and hypertension thus suggesting that HAART has a significant direct impact on blood pressure. In contrary, other authors like [14, 20] reported no effect of antiretroviral therapy on blood pressure. The data generated by various authors that are mentioned above show clearly the conflicting results in the association between HAART and hypertension. The type of antiretroviral therapy or HAART regimens used in the treatment of participants enrolled in the different studies could partially explain the conflicting results generated in different studies. In our study for instance, 54.91% of PLWHIV were on TDF/3TC/EFV, 19.20% on AZT/3TC/NVP, 14.73% on TDF/3TC/ATV/r, 6.70% on AZT/3TC/ATV/r and 2.68% on TDF/3TC/NVP. The choice of control groups is another factor that could also explain the conflicting results. For example, Gazzaruso *et al*. (2003) [14] compared HAART-treated HIV-positive persons with blood donor controls and found a higher blood pressure in the first group of participants. Other studies used sick HIV-positive treatment-naïve patients and patients from a medical out-patient clinic as the control group [16]. Ideally, the control group should be recruited from the same population from which the case arose as seen in the study of Serberg *et al*. 2005 [18]. All participants in our study were recruited from the same health facility.

Our study showed that TDF/3TC/EFV, AZT/3TC+EFV, AZT/3TC/EFV and TDF/3TC+NVP were associated to hypertension. This might be due to the presence of Tenofovir and Lamivudine in the different regimens. Our results are in line with those of [21], showing that treatment regimens containing Tenofovir/Lamivudine were associated with an increased risk of developing elevated blood pressure compared to those containing Zidovudine/Lamivudine. In another study Gallant *et al*. (2005) [22] showed that treatment with Tenofovir is associated with declined renal function that could lead to elevation of blood pressure. Furthermore, Khalsa A *et al*. in 2004 [23] reported that Zidovudine monotherapy is associated to reduce hypertensive risk among women. In our study where triple therapy containing either NNRTI and NRTI or NNRTI's and a PI were used, the impact of different classes of drugs or different drugs (ART) could not be examined since each HAART regimen was made up of three ART and at least two classes of drugs. However it is important to point out that, PLWHIV treated with HAART containing Atazanavir did not show any association with HTN. This is in agreement with results of Crane *et al.* (2006) which showed that people receiving Atazanavir-based regimens had a lower risk of developing elevated blood pressure compared with patients receiving lopinavir/ritonavir. The absence of association might be due to the presence of Atazanavir in the regimen. The association between the different ART or HAART regimens with HTN might depend on the type of ART or HAART used by the participants. Our study was limited by its cross-sectional design that could probably restrict any influence about causality. This study was conducted in the Bamenda regional hospital (BRH) in Cameroon and generalizability of results to other hospitals of other regions may not be possible. We did not also include HIV-negative controls like in other studies [14, 24]. We did not also distinguish primary from secondary hypertension. Our study did not control for potential confounders such as diabetes, renal disease and dyslipidaemia. Our findings reporting that TDF/3TC/EFV, AZT/3TC+EFV, AZT/3TC/EFV, and TDF/3TC+NVP were associated to hypertension and that HAART regimens containing Atazanavir were not associated to hypertension warrant further investigation ideally in the context of a randomized controlled trial.

## Conclusion

This study showed that the prevalence of hypertension in PLWHIV on HAART was twice that of PLWHIV who were not on HAART (HAART-naïve). Our result shows that PLWHIV and who are on HAART were more likely to have hypertension than those who are not on HAART. They show also a significant association between HAART and HTN. The treatment regimens TDF/3TC/EFV, AZT/3TC+EFV, AZT/3TC/EFV and TDF/3TC+NVP were associated to hypertension whereas AZT+3TC+ATV/r and TDF+3TC+ATV/r were not associated to hypertension. The high prevalence of hypertension, a known cardiovascular risk factor combined to the risk factor of metabolic disorders related to HAART are worrisome and should be monitored periodically and treated when necessary.

### What is known about this topic

- Other researchers from different areas carried out research on the prevalence of HTN between patients on HAART and HAART-naïve people and they had conflicting results in others the prevalence was higher in the HAART group than the HAART-naïve group and they found a significant difference between the two groups. Whereas in others there was no significant difference;
- Other researchers on other countries worked on the association between HAART and HTN and some say there is an association while others say there is no association;
- Another study was carried out to find out which particular therapy is associated to hypertension.

### What this study adds

- We found out that the prevalence of hypertension in the HAART group was two times higher than that of the HAART-naïve group and there was a significant difference between the two groups;
- We found a significant and positive association between HAART and hypertension(we are the first to find out this in Cameroon);
- We then went further to find out the prevalence of hypertension in the different HAART regimens and we found that the highest prevalence were in clients who took TDF/3TC/EFV and the lowest were in clients who took AZT+3TC+ATV/r; in our study, since all of our clients were on triple therapy it was not possible to assess the association that exist between each regimen and hypertension. But our study found out that TDF/3TC/EFV, AZT/3TC+EFV and TDF/3TC+NVP were associated to hypertension while regimens containing Atazanavir were not associated to hypertension (We are the first to find this in Cameroon).

## Competing interests

The authors declare no competing interests.
